# Mitochondrial D-loop mutations and deletion profiles of cancerous and noncancerous liver tissue in hepatitis B virus-infected liver

**DOI:** 10.1038/sj.bjc.6602496

**Published:** 2005-03-22

**Authors:** N M Wheelhouse, P B S Lai, S J Wigmore, J A Ross, D J Harrison

**Affiliations:** 1 Cell Injury and Apoptosis Group, Edinburgh MRC Centre for Inflammation Research, College of Medicine and Veterinary Medicine, University of Edinburgh, Teviot Place, Edinburgh EH8 9AG, UK; 2 Department of Surgery, Division of Hepato-biliary and Pancreatic Surgery, Chinese University of Hong Kong, Hong Kong SAR, China

**Keywords:** mitochondrial DNA, inflammation, viral hepatitis, tumorigenesis

## Abstract

The largest single underlying cause of hepatocellular carcinoma (HCC) worldwide is hepatitis B virus (HBV) infection. Hepatitis B virus increases cellular oxidative stress and the development of HCC occurs after a long latency period. The study was carried out to determine whether mitochondrial DNA abnormalities were associated with HCC in individuals with HBV. The frequency of mutation and deletion of specific areas of the mitochondrial genome in tumour and matched normal tissue of patients with HBV infection was investigated in the current study. The percentage of control subjects harbouring D-loop mutations was 11%, which was significantly lower than that observed in both the noncancerous (49%, *P*=0.033) and tumour tissue (59%, *P*=0.014) of patients with HCC. In contrast, the number of cases in which the common 4977 bp deletion of the mitochondrial genome was detected was significantly greater in control liver and noncancerous liver tissue of subjects with HCC (100 and 95%, respectively) than in cancerous liver tissue (28%, *P*<0.001). These observations suggest that the inflammatory process contributes to the rate of mitochondrial mutations. However, the lower frequency of the large deletion in cancerous tissue suggests that there is selection against either mitochondria, which harbour large deletions, or against cells that contain these mitochondria during hepatocarcinogenesis.

Hepatocellular carcinoma (HCC) is the fifth most frequent cancer worldwide and is the third highest cause of cancer-related death (Block *et al*, 2003). Unlike many forms of cancer, more than 95% of HCC cases arise upon a background of persistent inflammatory disease. Although the disease aetiologies are diverse, hepatitis B virus (HBV) and hepatitis C virus (HCV) are implicated in the majority of cases (Waris and Siddiqui, 2003).

Chronic infection with either HBV or HCV results in the production of reactive oxygen species (ROS) in the liver, leading to an increase in potentially mutagenic DNA lesions (Waris and Siddiqui, 2003). Mitochondrial DNA is more susceptible to sustained DNA damage compared with nuclear DNA due to the lack of histones and decreased capacity for DNA repair (Kang and Hamasaki, 2002). While genomic DNA is complex, the human mitochondrial genome is only 16.5 kb in length, encoding 13 polypeptides of the respiratory chain, 22 transfer RNAs and two rRNAs (Taanman, 1999). The displacement loop (D-loop) region is the major control site for mtDNA expression containing the origin of replication for the heavy DNA strand and the major promoters of transcription (Taanman, 1999). Mutations within the D-loop region have been detected in a variety of cancers including oesophageal (Miyazono *et al*, 2002), gastric (Máximo *et al*, 1999), thyroid (Máximo *et al*, 2002), breast (Richard *et al*, 2000) and HCC in HCV-infected individuals (Nishikawa *et al*, 2001).

Although various deletions of the mitochondrial genome have been reported, the 4977 bp deletion is the most widespread and has been associated with ageing in tissues (Kowald, 1999). While increases in the frequency of the 4977 bp deletion have been demonstrated in tumours, the levels in HCC of subjects with HCV is thought to be reduced (Kotake *et al*, 1999).

We hypothesised that increased oxidative stress associated with HBV infection in individuals with HCC would lead to an increased burden of mitochondrial DNA mutation. We therefore sequenced a small portion of the hypervariable D-loop region of mitochondria from individuals with HCC in matching tumour and nontumour liver tissue to determine the frequency of mitochondrial mutations. In addition, we investigated the frequency of deletions in noncancerous and tumour tissue to help understand the mechanisms that lead to tumorigenesis.

## MATERIALS AND METHODS

### Tissue specimens

We collected 124 liver specimens from 62 ethnic Chinese patients who underwent surgery at the Department of Surgery, Chinese University of Hong Kong. These included 59 HCC and corresponding noncancerous but cirrhotic tissue from individuals with chronic HBV, and an additional three paired samples from individuals with HCV and HCC. In addition, we obtained a further nine control samples of normal liver from patients undergoing partial hepatic resection with diseases other than HCC, usually colorectal metastases. All samples were obtained with appropriate local ethical committee approval and informed consent was obtained from recruited patients.

### Analysis of mutations

DNA was extracted from tissue samples by the QiaAmp DNA micro kit (Qiagen, USA). Each DNA sample (100 ng) underwent PCR amplification with specific primers designed using the Primer 3 program (Rozen and Skaletzky, 1996) to amplify a 334 bp region of the D-loop (forward primer: 5′-gagctctccatgcatttggt-3′; reverse primer: 5′-tctttgtttttggggtttgg-3′). Cycle conditions were as follows: an initial denaturation step of 94°C for 5 min; two cycles of 94°C for 45 s, 64°C for 1 min 15 s, 70°C for 30 s; 35 cycles of 94°C for 45 s, 58°C for 1 min 15 s, 70°C for 30 s; final extension step of 70°C for 10 min. Sequencing was performed with an ABI 377 gel-based sequencing machine after labelling with the BigDye terminator cycle sequencing kit 3.1 on a GeneAmp PCR system 9700 (Applied Biosystems, Foster City, CA, USA), using the same PCR primers.

### Analysis of the common deletion

The method employed was essentially similar to that described previously (Máximo *et al*, 2002), using the internal primers (MITIN: forward, 5′-ctgagccttttaccactccag-3′, reverse 5′-ggtgattgatactcctgatgcg-3′) and external primers spanning the deleted fragment (MITOUT: forward 5′-cccaactaaatactaccgtatgg-3′, reverse 5′-ggctcaggcgtttgtgtatgat-3′) as described (Máximo *et al*, 1999). Cycle conditions were as follows: an initial denaturation step of 95°C for 15 min; 40 cycles of 94°C for 30 s, 58°C for 30 s, 72°C for 30 s; final extension at 72°C for 2 min. Products were resolved on a 1.6% agarose gel, stained with ethidium bromide and visualised under UV illumination.

### Statistics

The frequency of mutations and deletions in samples were analysed by nonparametric Mann–Whitney *U*-test. Associations between the presence of either gene deletion or mutations and clinicopathological markers of tumour behaviour such as capsular invasion and tumour stage were studied by univariate analysis and by multivariate analysis using a Cox proportional hazards model in which a time constant was set and where either the presence of gene deletion or mutation in the tumour was set as the event. Statistical analysis was performed using SPSS 12.0, Chicago, IL, USA).

## RESULTS

### Patient characteristics

The clinical characteristics of the 62 patients composing the study group are given in Table 1. The majority were male and infected with HBV. Only six of the patients had normal underlying nontumour liver tissue, the remainder had either fibrosis or cirrhosis. Four patients had major vascular invasion by tumour and a further eight patients had microvascular invasion. There were no statistical associations between the presence of either mitochondrial D-loop deletions or mutations and the clinicopathological features of aggressiveness of the HCCs examined.

### PCR and sequencing

**Figure 1:**
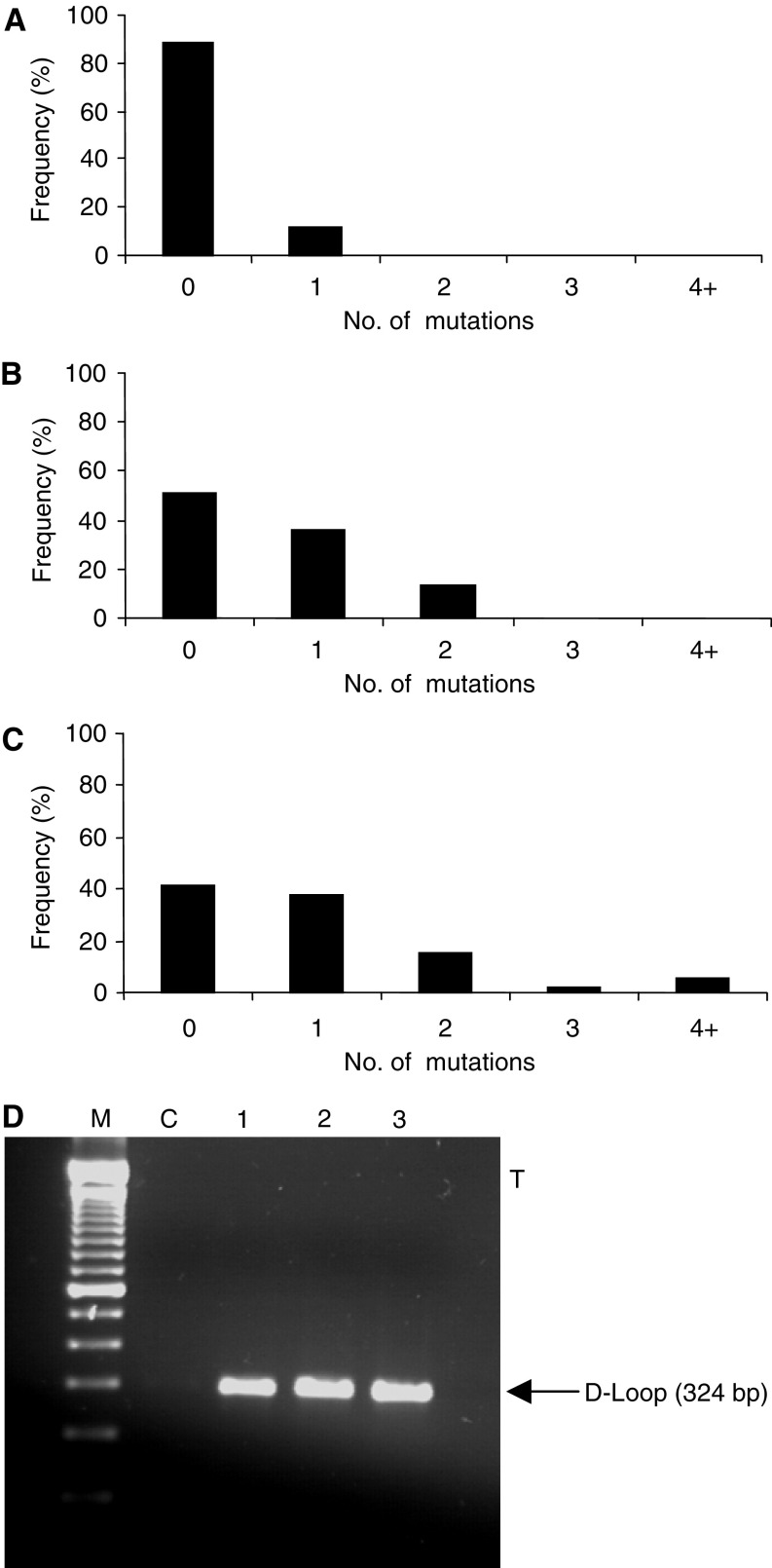
Frequency of mutation in the D-loop region of (**A**) control tissue, (**B**) noncancerous liver tissue and (**C**) HCC tumour tissue. (**D**) shows a typical gel. M: markers. Lane C shows the negative control. Lanes 1, 2 and 3 show PCR products derived from control, noncancerous and tumour liver tissue, respectively.

**Figure 2:**
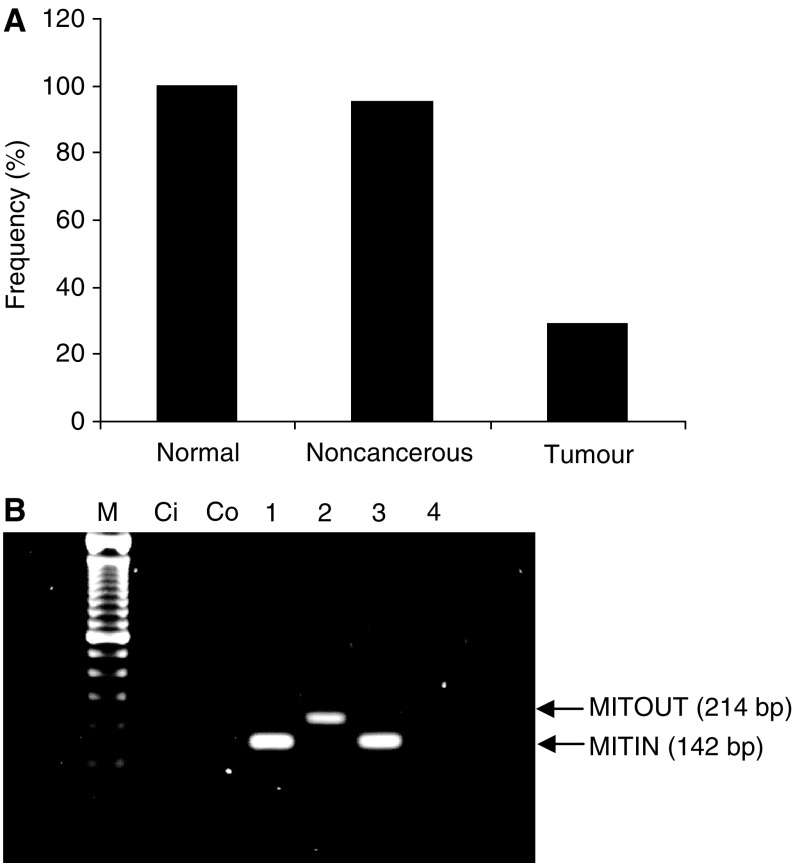
(**A**) Frequency of the 4977 bp deletion in control tissue, noncancerous tissue and HCC liver tissue. (**B**) A typical gel. Lanes Ci and Co are the negative control lanes for MITIN and MITOUT PCRs. Lanes 1 and 3 show the 142 bp PCR products derived from using the MITIN internal control primers. Lanes 2 and 4 show the presence and absence of the deleted band, respectively (214 bp).

For mutation analysis, we successfully sequenced DNA from 61 of the 62 sample pairs of cancerous and noncancerous liver tissue, and all control samples (Figure 1D shows a representative gel). For deletion analysis, all 62 sample pairs and all controls were successfully analysed. The presence of the 4977 bp deletion was determined by the presence of a 214 bp band (Figure 2B). Routinely using the MITOUT primers, a faint nonspecific band could be detected in both positive and negative samples. After sequencing, this was found to be a unidirectional PCR product from the forward primer, terminating outside the deleted region and was also used as an additional internal PCR control. In addition, both specific products from the MITIN and MITOUT PCRs were sequenced to validate the assay.

### Mutations increase in both cancerous and noncancerous liver tissue of patients with HBV

Comparison of mutations was performed against an mtDNA sequence, Genbank (accession no. JO1415). All samples contained a T → C transition at nucleotide 489, a C insertion between 311 and 312 and all but one sample pair exhibited a G → A transition at nucleotide 263. These have been reported elsewhere as polymorphisms (Andres *et al*, 1999) and were not included in subsequent analysis. The frequency of subjects harbouring D-loop mutation, which is summarised in Figure 1, was significantly greater in the liver tissue of subjects with HCC compared with normal control cases (59 *vs* 11%, *P*=0.014), and noncancerous tissue of patients with HCC compared with control tissue (49 *vs* 11%, *P*=0.033). On the whole, the same mutations (if present) observed in noncancerous tissue were also present in tumour tissue from the same individual, although additional mutations were observed in some of the tumour samples. However, there was no significant increase in the frequency of mutation in liver tumour tissue compared to paired noncancerous liver tissue from the same patient (*P*=0.41).

### Frequency of mtDNA deletion is reduced in cancerous tissue

In contrast to the results obtained for mtDNA mutations, the prevalence of deletion was significantly lower in HCC tissue compared to noncancerous tissue (28 *vs* 95%; *P*<0.001). The frequency of deletion within control normal liver tissue was similar to that obtained for noncancerous liver tissue of patients with HCC (100 *vs* 95%) (Figure 2).

## DISCUSSION

We have demonstrated that mitochondrial DNA abnormalities are common in the D-loop of patients with HCC upon a background of HBV. The D-loop region is a major control site for mtDNA replication containing the leading origin of replication and the major promoters required for transcription. HBV infection, in terms of patient numbers, is a more important clinical disease worldwide and is responsible for nearly 60% of HCC cases (Lai *et al*, 2003). While mitochondrial mutations have been demonstrated in HCC, previous studies have been carried out on individuals with hepatitis C (Nishikawa *et al*, 2001; Lee *et al*, 2004; Kubo *et al*, 2003).

The 4977 bp deletion is deleterious, affecting much of complex I of the electron transport chain, yet due to the number of mitochondria and multiple copies of mtDNA in each mitochondrion only 0.004% of liver mtDNA is thought to be affected in normal individuals (Kotake *et al*, 1999). The incidence of the 4977 bp deletion in liver increases with the age of subjects (Fukushima *et al*, 1995) and with chronic alcohol abuse (Mansouri *et al*, 1997). In our series, the mean age was 55 years (±11.5), and it is therefore not surprising to detect the common 4977 bp deletion in 95% of the noncancerous tissue samples and also the control subjects. Yet, while ageing is associated with an increased incidence of mtDNA deletion, we found a lower prevalence of deletions in the cohort of HCC.

In the current study, no matched blood samples were available to characterise unknown polymorphisms in this patient cohort. Nevertheless, given the similaritities in the frequency of D-loop variants observed in the current study and those observed in previous studies, albeit on individuals with HCC from different disease aetiologies (Nishikawa *et al*, 2001; Kubo *et al*, 2003), indicate that these data give an accurate representation of mutation frequency within the sequence of mtDNA analysed.

We observed a decrease in the frequency of the common 4977 bp mtDNA deletion in cancer compared to noncancerous liver tissue. This is in apparent agreement with two previous studies, the first of which demonstrated a decrease in deletion frequency between normal, cirrhotic and tumour tissue (Kotake *et al*, 1999), and the second of which indicated a decrease in the deletion level in tumour compared to noncancerous in males but not females (Yin *et al*, 2004). In our cohort of patients who had tumours containing the deletion, all were male; however, as only seven of 62 of our subjects were female, it would be difficult to make comparisons between the sexes. In a third study, mtDNA deletions could not be detected in either noncancerous or tumour tissue (Nishikawa *et al*, 2001); however, it is uncertain whether this is due to methodology or if this was due to the differences in the effects of underlying HCV to other disease aetiologies.

We observed in three tumours a deletion of the c-tract region of the D-loop, which was not apparent in the paired noncancerous tissue. Replication of mtDNA uses DNA polymerase *γ*, which has a reduced fidelity for homopolymeric sequences, making this particular region highly susceptible to mutation and deletion (Copeland *et al*, 2003). In a setting of increased cellular and thus mitochondrial replication, there is an increased opportunity for uncorrected errors to be introduced. This suggests that while mitochondria containing large deletions in the encoding regions of DNA are eliminated during transformation, small deletions around the regulatory D-loop may provide a selective advantage. Thus, abnormalities within mitochondrial DNA could have important consequences in terms of mitochondrial replication, as well as being an indicator of more widespread DNA damage in the cell. Indeed, it has been demonstrated that mitochondria of tumour cells selectively proliferate when tumour cells are fused with normal cells (Polyak *et al*, 1998).

This is the first report to our knowledge that has made observations on mtDNA mutations and deletions in patients with HBV, the largest single underlying cause for HCC worldwide. The presence of mtDNA deletions are thought to be indicative of tissue ageing, yet in the apparently normal tissue of patients with HCC, they exist in conjunction within mitochondria containing homoplasmic mutations of the D-loop. This observation of deletions in conjunction with mutations indicates that mutation of the D-loop occurs at a relatively early stage in HBV infection. The development of HCC only occurs after a long latency period of 20–30 years after the initial HBV infection, and these observations would indicate that mtDNA mutations occur independently of neoplastic transformation. Conversely, the reduction in the frequency of the common deletion in liver tumours indicates that there is an active selection pressure against the presence of mtDNA with large deletions, or more controversially that a population of liver cells do not undergo the typical ageing response and subsequently form the liver tumour. As yet, there is little evidence to support these hypotheses; however, it has been shown recently that mitochondrial DNA copy number is reduced in tumours (Lai *et al*, 2003; Yin *et al*, 2004), possibly indicating an active selection process during neoplasia. Yet, while mutations and deletions appear to be relatively common in these patients and may be related to carcinogenesis, they do not appear to be related to an aggressive phenotype on the basis of their clinical and pathological behaviour.

In summary, our data clearly demonstrates that mtDNA mutations are present in a large proportion of HCC upon a background of HBV infection, but deletions are rare. Moreover, the observation that deletions coexist with mutations in noncancerous but not HCC tissue may indicate that there is either (i) a commitment to form HCC occurs at an early stage before ageing effects are noted or (ii) a strong selection pressure during carcinogenesis in favour of cells containing mitochondria with no deletions. This may account for the long latency period previously noted.

## Figures and Tables

**Table 1:** Characteristics of patients with HCC and their tumours

	**Number/median (range)**	**Percentage**
Age (years)	55 (32–78)	

*Gender*		
Male	55	(89%)
		
HBV	58	(94%)
HCV	5	(8%)
		
*Nontumour liver tissue*		
Normal	6	(10%)
Fibrosis	19	(31%)
Cirrhosis	37	(60%)
		
*AJCC tumour stage*		
1	2	(3%)
2	39	(63%)
3	15	(24%)
4	6	(10%)
		
Tumour maximum size (cm)	3.5 (1.6–11.9)	
		
*Capsular invasion*		
Y	34	(50%)
N	18	(29%)
No capsule	10	(21%)
		
*Microvascular invasion*		
Y	8	(13%)
N	54	(87%)
		
Serum alpha fetoprotein (U/l)	96 (2–699 800)	

HCC=hepatocellular carcinoma; HBV=hepatitis B virus; HCV=hepatitis B virus.
